# A Challenge for the Seed Mixture Refuge Strategy in Bt Maize: Impact of Cross-Pollination on an Ear-Feeding Pest, Corn Earworm

**DOI:** 10.1371/journal.pone.0112962

**Published:** 2014-11-19

**Authors:** Fei Yang, David L. Kerns, Graham P. Head, B. Rogers Leonard, Ronnie Levy, Ying Niu, Fangneng Huang

**Affiliations:** 1 Department of Entomology, Louisiana State University Agricultural Center, Baton Rouge, Louisiana, 70803, United States of America; 2 Macon Ridge Research Station, Louisiana State University Agricultural Center, Winnsboro, Louisiana, 71295, United States of America; 3 Monsanto Company, St. Louis, Missouri, 63167, United States of America; 4 Dean Lee Research Station, Louisiana State University Agricultural Center, Alexandria, Louisiana, 71302, United States of America; Federal University of Viçosa, Brazil

## Abstract

To counter the threat of insect resistance, *Bacillus thuringiensis* (Bt) maize growers in the U.S. are required to plant structured non-Bt maize refuges. Concerns with refuge compliance led to the introduction of seed mixtures, also called RIB (refuge-in-the-bag), as an alternative approach for implementing refuge for Bt maize products in the U.S. Maize Belt. A major concern in RIB is cross-pollination of maize hybrids that can cause Bt proteins to be present in refuge maize kernels and negatively affect refuge insects. Here we show that a mixed planting of 5% nonBt and 95% Bt maize containing the SmartStax traits expressing Cry1A.105, Cry2Ab2 and Cry1F did not provide an effective refuge for an important above-ground ear-feeding pest, the corn earworm, *Helicoverpa zea* (Boddie). Cross-pollination in RIB caused a majority (>90%) of refuge kernels to express ≥ one Bt protein. The contamination of Bt proteins in the refuge ears reduced neonate-to-adult survivorship of *H. zea* to only 4.6%, a reduction of 88.1% relative to larvae feeding on ears of pure non-Bt maize plantings. In addition, the limited survivors on refuge ears had lower pupal mass and took longer to develop to adults.

## Introduction

Transgenic crops (maize and cotton) expressing *Bacillus thuringiensis* (Bt) proteins were planted on >72 million hectares for pest control in the world in 2013 [1]. In the U.S. alone, nearly 30 mha or 76% of the field maize was planted to Bt maize in the same year [2]. Field performance of Bt crops, in general, has been very effective against the target insect pests [3]–[7]. However, the intensive use of Bt crops places a high selection pressure on the target pest populations that could lead to the rapid evolution of resistance [8]–[14]. To delay resistance development, a ‘high dose/refuge’ strategy has been adopted for planting Bt maize in the U.S. and several other countries [8], [15]–[17]. This strategy is based on the concept that Bt-susceptible insects produced in refuge areas will mate with the rare resistant homozygous individuals that might emerge from the Bt crop. If the frequency of resistance is low enough, most offspring will be heterozygous and thus should be killed by the high-dose Bt plants. Therefore, resistance allele frequencies in field populations should remain low for a long period of time. Before 2010, the refuge was required to be arranged in a structured form that was implemented as blocks or strips of non-Bt crops [16], [17]. Since 2010, a seed mixture approach (also called refuge-in-the-bag or RIB) of 5∶95% (nonBt: Bt maize seeds) [18] has been approved and adopted by growers for planting pyramided Bt maize products in the U.S. Maize Belt [15], [19], [20]. Pyramided Bt maize hybrids contain two or more Bt genes targeting the same pest species [21]–[23]. Due to differences in the predominant pests from the Maize Belt, and particularly the overwintering of the ear-feeding corn earworm, *Helicoverpa zea* (Boddie) [19], [20], [24]–[26], a RIB strategy has not been approved in the southern U.S. [15]. A major concern in implementing RIB is cross-pollination of maize hybrids that can cause Bt proteins to be present in refuge maize kernels in seed mix plantings [27]–[29]. The Bt protein contamination in RIB could negatively affect (e.g., survival, growth, and development) refuge insects, if they are ear feeders. However, prior to this study, the intensity of Bt protein contamination in RIB and its associated effects on refuge populations of ear feeders under real field conditions have not been investigated. Argument over the effectiveness of RIB strategies for resistance management has been a hot topic for two decades [19], [27], [29]–[32].

*H. zea* is a major target species of pyramided Bt maize in both North and South America and its damage to maize is primarily caused by larvae feeding on ear kernels [33]. Thus, the RIB-*H. zea* system provides an excellent model to study the effect of cross-pollination on refuge populations of ear feeding species. In 2012–2013, field and laboratory studies were conducted to assess the intensity of Bt protein contamination in a RIB planting of 5% non-Bt and 95% Bt maize containing the SmartStax trait and the corresponding effect of the cross-pollination on survival, growth, and development of *H. zea*. SmartStax is a common pyramided Bt maize product that expresses the Cry1A.105, Cry2Ab2 and Cry1F proteins targeting above-ground lepidopteran species including *H. zea*. The results show that the 5∶95% RIB approach is not effective for providing refuge for *H. zea*. Our study is timely given growing concerns over the resistance management for Bt crops. It is also an important guide for regulators in making science-based decisions regarding the suitability of seed mixture strategies for different regions.

## Results and Discussion

### Bt protein contamination of refuge kernels in RIB plantings

Qualitative ELISA tests (Figure S1) showed that all 150 individual kernels sampled from 30 ears of pure Bt maize plantings in three field trials expressed all four Bt protein groups (six Bt proteins) in SmartStax (Table 1). Similarly, all kernels from 30 ears sampled from pure non-Bt plantings were free of Bt protein expression, suggesting that there was no cross-pollination among the trial fields. However, cross-pollination within RIB fields resulted in most (94.4%) refuge ear kernels expressing at least one Bt protein. Frequency of Bt protein expression in refuge kernels was consistent among the three field trials with an average of 55.1, 29.5, 14.0, and 5.1% kernels expressing one to four groups of Bt proteins, respectively (Table 1). Based on the Bt expression recorded in refuge kernels, χ^2^ tests showed that the four protein groups segregated independently in all three trials with only a few exceptions (Table 1, Table S1). Limited by the techniques, the concentration of each Bt protein in individual kernels was not measured. Studies have shown that production of Bt proteins is typically dominant in Bt plants and thus concentration in the refuge kernels is expected to have been high [27], [34]. In addition, the strong bands exhibited in the ELISA strips (Figure S1) also indicated that the Bt protein expression levels were not low.

**Table 1:** Percentage of individual kernels expressing Bt proteins in SmartStax maize in pure Bt, pure non-Bt, and RIB plantings*.

Bt protein group	Pure Bt	Pure non-Bt	RIB refuge
Cry1A/Cry2A	100	0	49.2
Cry3B	100	0	74.4
Cry1F	100	0	45.1
Cry34/35Ab	100	0	51.8
Mean ± sem	100±0.0	0.0±0.0	55.1±6.6
Cry1A/Cry2A+Cry3B	100	0	38.0
Cry1A/Cry2A+Cry1F	100	0	22.1
Cry1A/Cry2A+Cry34/35Ab	100	0	12.8^s^
Cry3B+Cry1F	100	0	36.4
Cry3B+Cry34/35Ab	100	0	42.1
Cry1F+Cry34/35Ab	100	0	25.6
Mean ± sem	100±0.0	0.0±0.0	29.5±4.6
Cry1A/Cry2A+Cry3B+Cry1F	100	0	17.4
Cry1A/Cry2A+Cry3B+Cry34/35Ab	100	0	10.3^s^
Cry1A/Cry2A+Cry1F+Cry34/35Ab	100	0	6.2^s^
Cry3B+Cry1F+Cry34/35	100	0	22.1
Mean ± sem	100±0.0	0.0±0.0	14.0±3.6
Cry1A/Cry2A+Cry3B+Cry1F+Cry34/35Ab	100	0	5.1±1.8
Negative for Bt protein expression	0.0±0.0	100±0.0	5.6±1.9

*Means in the table were overall means across three trials (Table S1). In each field trial, 5 individual kernels per ear with 10 ears were examined for ears of pure Bt maize plantings (Pure Bt); 25 kernels per ear with 10 ears were tested for ears of pure non-Bt maize plantings (pure non-Bt); and for refuge ears in RIB (RIB refuge), 5 individual kernels per ear with 13 ears were assayed.

s Observed frequencies do not fit the assumption of independent segregation in χ^2^-tests (*P*<0.05) (Table S1).

It has been commonly assumed that, for a particular gene in pure Bt maize plantings of a maize hybrid (F_1_), 25% F_2_ kernels should be homozygous and 50% should be hemizygous for the Bt allele, while the remaining 25% should not express the Bt protein [27], [28], [35], [36]. Contrary to this common assumption, our results suggest that alleles of all Bt genes in SmartStax are likely homozygous in the two parents of the F_1_ maize hybrids. Similarly in another independent study, we also found that 100% F_2_ maize kernels of a pure-planted Bt maize hybrid containing the Agrisure Viptera 3111 trait expressed both the Vip3A and Cry1Ab proteins (Yang et al. unpublished data). The results of these studies suggest that the ‘homozygous’ property of Bt genes may commonly exist in different Bt maize products.

### Effects of Bt protein contamination in RIB on refuge populations of *H. zea*

**Figure 1:**
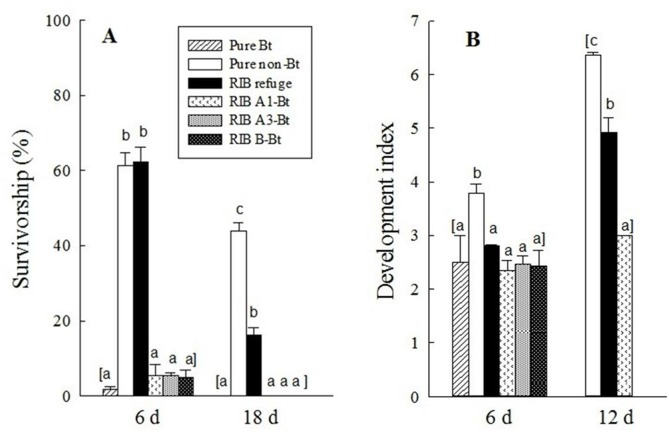
In-field observation on survivorship (A), and development (B) of *H. zea* on ears of SmartStax Bt and non-Bt maize plants in three planting patterns. Detailed data are reported in Table S2. Pure Bt: pure Bt maize planting; Pure non-Bt: pure non-Bt maize planting; RIB refuge: the refuge plants in the RIB planting; A1-Bt: the Bt plants immediately adjacent and within the same row as the refuge plant in RIB planting; A3-Bt: the 3^rd^ Bt plants on both sides of the refuge plant in the same row in RIB planting, and B-Bt: the closest Bt plants on both sides of the refuge plant in the two adjacent rows in RIB planting. Insect development was converted to development index: 1 = 1^st^ instar, 2 = 2^nd^ instar, …, 6 = 6^th^ instar, 7 = pupal stage. Sample size for measuring survivorship was 240 larvae for RIB and 120 larvae for pure Bt and pure non-Bt. Sample size for determining larval development on pure non-Bt and RIB refuge was 55–150 larvae and on Bt plants was 1–13 larvae. Mean values within an observation time followed by a different letter were significantly different (Tukey’s HSD test, α = 0.05).

**Figure 2:**
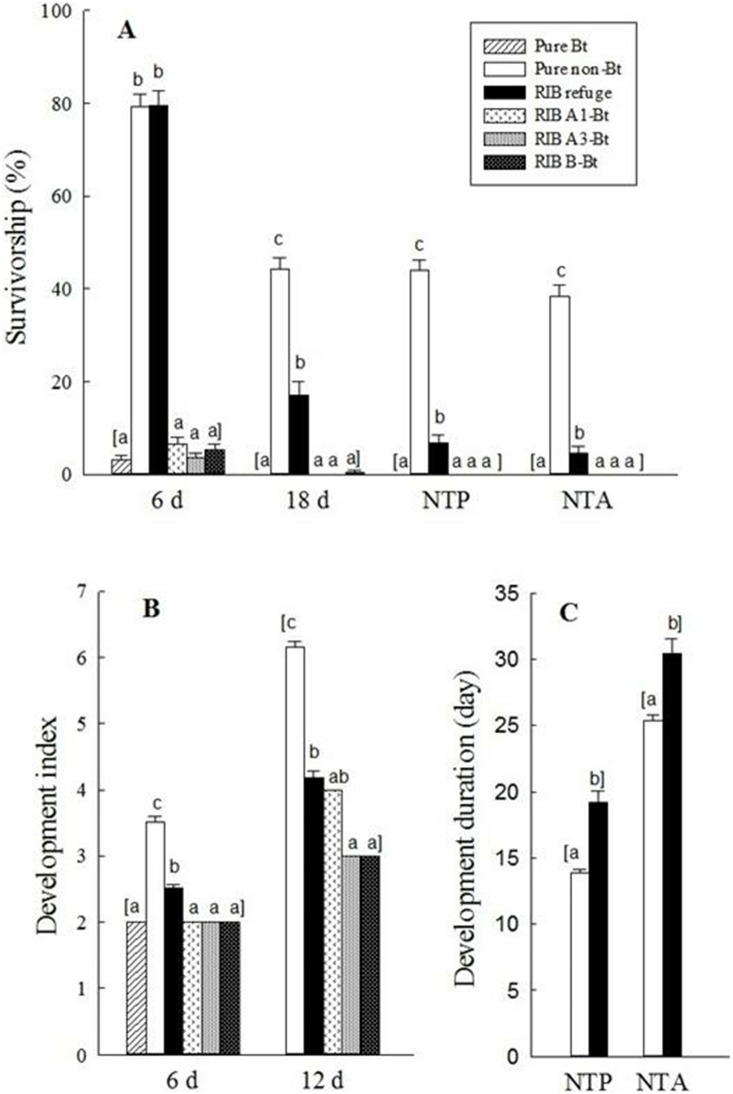
Lab assay on survivorship (A), development (B), and development duration (C) of *H. zea* on ears of SmartStax Bt and non-Bt maize plants in three planting patterns. Detailed data are reported in (Table S3–S4). Pure Bt: pure Bt maize planting; Pure non-Bt: pure non-Bt maize planting; RIB refuge: the refuge plants in the RIB planting; A1-Bt: the Bt plants immediately adjacent and within the same row as the refuge plant in RIB planting; A3-Bt: the 3^rd^ Bt plants on both sides of the refuge plant in the same row in RIB planting, and B-Bt: the closest Bt plants on both sides of the refuge plant in the two adjacent rows in RIB planting. Insect development was converted to development index: 1 = 1^st^ instar, 2 = 2^nd^ instar, …, 6 = 6^th^ instar, 7 = pupal stage. NTP: neonate-to-pupa; NTA: neonate-to-adult. Means were calculated based on four independent assays (treated as a random factor). Sample size for each treatment mean was based on 300 larvae for measuring survivorship. Sample size for determining larval development was 122–239 larvae for pure non-Bt and RIB refuge, and 1–19 larvae for Bt plants. Mean values within an observation time followed by a different letter were significantly different (Tukey’s HSD test, α = 0.05).

**Figure 3:**
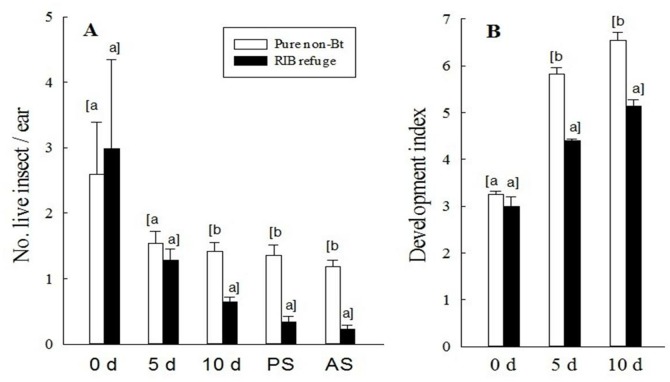
Field-plus-lab assay on occurrence (A), and development (B) of *H. zea* on ears of RIB refuge and pure non-Bt plants. Pure non-Bt: pure non-Bt maize planting; and RIB refuge: the refuge plants in the RIB planting. Insect development was converted to development index: 1 = 1^st^ instar, 2 = 2^nd^ instar, …, 6 = 6^th^ instar, 7 = pupal stage. PS: pupal stage; AS: adult stage. 0 d = the day that ears were sampled from fields. Means were calculated based on three independent assays (treated as a random factor). Sample size for each treatment mean was 160 ears for larval occurrence. Sample size for determining larval development was 104–479 larvae. Mean values within an observation time followed by a different letter were significantly different (Tukey’s HSD test, α = 0.05).

Multiple field trials and laboratory assays showed that the high levels of Bt protein contamination in refuge ears in RIB described above significantly affected larval survival, growth, and development of *H. zea*. The overall results were consistent across three study methods including in-field observation, lab assay and field-plus-lab assay (Figures 1–3), as well as across all trials within each study method (Tables S2–S5). SmartStax is very effective against *H. zea*. In both in-field observation and lab assays, no *H. zea* neonates developed to the pupal stage on ears of Bt plants either in pure Bt maize plantings or RIB (Figures 1A & 2A). Both study methods also showed that Bt protein contamination in refuge ears did not significantly affect larval survival at the early insect stages (e.g., at 6 d after release of neonates). For example, after 6 d of neonate release, larval survivorship for in-field observation was 62.3% on refuge ears and 61.2% on pure non-Bt ears, and these values in lab assay were 79.4 and 79.6%, respectively. However, the Bt protein contamination delayed larval development by approximately one instar for both study methods. After 12 d as well as in subsequent observations, both larval survivorship and development were affected considerably. For example, at 18 d, survivorship on pure non-Bt maize ears was 43.9% for in-field observation and 44.2% in lab assay, while on refuge ears it was only 16.2 and 17.1%, respectively. Similarly, compared to pure non-Bt maize ears, larval development after 12 d on refuge ears was significantly delayed by 1.5- and 2.0-instar for the in-field observations and lab assays, respectively (Figures 1B & 2B).

Pupation and adult emergence were not measured for the in-field observation because mature larvae of *H. zea* moved out from the ears and dropped into soil for pupation [37]. In the lab assay, 43.9 and 38.3% neonates on pure non-Bt maize ears successfully developed to pupae and adults, respectively, while these values on refuge ears were only 6.7 and 4.6%, which corresponded to a reduction of 84.7% for pupation and 88.1% for adult emergence (Figure 2A). In addition, the limited survivors on refuge ears had significantly lower pupal mass (284.3 mg/pupa) and longer developmental time to become pupae (19.2 d) or adults (30.4 d) compared to the pupal mass (413.5 mg/pupa) and developmental times (13.9 d to pupa and 25.4 d to adult) on pure non-Bt maize ears (Figure 2C, Table S4). The results suggest that far fewer susceptible insects will be produced from the 5% refuge in RIB plantings than by a 5% structured refuge.

To control for the effect of possible Bt protein degradation within the lab assay, a ‘field-plus-lab assay’ method was also employed. This method used field-collected ears containing naturally occurring early-stage larvae (3^rd^ to 4^th^ instars) of *H. zea*. Previous field studies have shown that natural occurrence of *H. zea* on refuge ears in RIB plantings was not affected by Bt protein contamination at the early larval stages (e.g. 3^rd^–4^th^ instars) [6]. This result led to the use of a field-plus-lab assay method to shorten the necessary laboratory assay duration so that the effect, if any, of Bt protein degradation in detached ears on the results could be minimized. Results of the field-plus-lab assay showed that at the time when ears were collected from field plants there were no significant differences in number of larvae per ear or larval development between pure non-Bt and RIB plantings (Figure 3). However, over time, the number of live *H. zea* on refuge ears decreased significantly and larval development on refuge ears was delayed significantly compared with the larvae on pure non-Bt maize ears. For example, at 10 d after ears detached from plants, the number of live larvae on refuge ears was reduced by 54.2% and larval development delayed by 1.5-instar compared to pure non-Bt maize ears. Ultimately, the Bt protein contamination reduced pupation by 75.0%, pupal mass by 22.7%, and moth emergence rate by 80.5% (Figure 3, Table S5). Results of the field-plus-lab assay validated that the 5∶95% RIB will be not effective in providing refuge populations for *H. zea*.

Previous reports indicated that Bt proteins could decrease with time in excised leaf tissue of cotton and maize, but the biological activity was maintained for at least several days [38]–[40]. We expected that the biological activity in detached maize ears should be maintained much longer than in detached leaf tissue because maize ears can be preserved considerably longer than leaf tissue. The similar results observed across the in-field observations, lab assays, and field-plus-lab assays suggest that the protocols used in the current study were appropriate. Nevertheless, if Bt degradation after ears are detached is significant, the effect of cross-pollination of intact plants on insect populations could be greater than that observed in this study. In addition, reproduction of many lepidopteran species is proportional to the nutrient reserves acquired during larval stages and is correlated with pupal weight [41]. Thus, the reduced pupal weight plus delayed development of *H. zea* feeding on refuge ears suggest that the cross-pollination could have additional effects on the adult reproduction.

Another concern with RIB plantings is that larval movement among Bt and non-Bt plants may hasten resistance evolution [18], [28], [42]–[44]. Larval movement among Bt and non-Bt plants could create sub-lethal exposure and promote build-up of resistance in target pest populations by increasing survival of the resistant heterozygotes or individuals carrying minor resistance alleles [45]. This could be true for both stalk borers and ear-feeders. Unfortunately, field data quantifying such effects are not available. Movement of susceptible larvae from non-Bt refuge plants to Bt plants in RIB plantings could also cause greater mortality to a susceptible population than in a structured refuge planting and result in a lower refuge population [30]. However, a recent study showed that the number of Bt-susceptible larvae of the sugarcane borer, *Diatraea saccharalis* (F.), a major target of Bt maize in the mid-southern U.S., on the refuge plants in a RIB planting was not reduced compared to the pure stand of non-Bt maize [45]. In addition, the delayed development and reduced pupal masses of the limited survivors of *H. zea* on the ears of refuge plants observed in the current study also suggest that even those larvae that stay and survive on the refuge plants without interplant movements can be affected by sublethal doses in RIB plantings.

In conclusion, results of the comprehensive field and laboratory studies suggest that RIB for Bt maize at the levels at which it is currently implemented in the U.S. Maize Belt is unlikely to provide adequate refuge populations for ear-feeding targets such as *H. zea*. Effective refuge strategies must be built upon appropriate analyses of all key pests and may require different approaches in different regions [32].

## Materials and Methods

### Ethics statement

The field trials described below were permitted by Louisiana State University Agricultural Center. The field work did not involve any endangered or protected species. No human participants, specimens or tissue samples, or vertebrate animals, embryos or tissues were involved in the study.

### Sources of maize and insects

Three Bt maize hybrids (DKC 61-21, DKC 55-09, and DKC 62-08, Monsanto, St. Louis MO) containing the SmartStax trait and two closely related non-Bt maize hybrids (DKC 61-22 and DKC 66-49, Monsanto, St. Louis MO) were used in this study. SmartStax contains Cry1A.105, Cry2Ab2 and Cry1F for controlling above-ground lepidopteran species, and Cry3Bb1 and Cry34Ab1/35Ab1 for managing below-ground rootworms, *Diabrotica* spp [23].

Laboratory populations of *H. zea* were established from feral larvae (∼100 individuals for each population) collected from non-Bt maize fields in Rapides Parish, Louisiana and Hidalgo County, Texas, USA. Field collected larvae were individually reared in 30-ml plastic cups containing a pre-mixed meridic diet (WARD’S Stonefly Heliothis diet, Rochester, NY). Pupae removed from the rearing cups were placed into ∼20 L mesh cages (Seville Classics, INC., Torrance, CA) containing ∼200 g vermiculite (Sun Gro, Pine Bluff, AR) and 10% honey water solution. The cages were then placed in growth chambers at 26.8°C, >90% RH and a 14∶10 h (L: D) photoperiod for adult emergence, mating, and oviposition. F_1_ neonates (<24 h old) from the field-collected *H. zea* were used, except where otherwise specified, in all field trials and laboratory bioassays in this study.

### Field planting

A total of three field trials were conducted in two locations in Louisiana, USA in 2012 (one trial) and 2013 (two trials). The 1^st^ (2012) and 2^nd^ (2013) trials were located in Franklin Parish (32° 08′N; 91° 41′W) in northeast Louisiana and the 3^rd^ trial was conducted in Rapides Parish (31° 10′ 35.99′'N; 92° 23′ 24.24′'W) in central Louisiana in 2013. Each trial consisted of three planting patterns: 1) pure stand of Bt plants, 2) pure stand of non-Bt plants, and 3) a RIB planting of 5% non-Bt (refuge) and 95% Bt plants. There was a distance of >300 m between fields, and no other maize plants at similar growth stages were planted within 300 m of the trial fields. The designed isolation should avoid any pollen contamination to the trial fields [46].

In each trial, there were ∼4000–6000 plants in 12 rows (with 102 cm between rows and 15.2 cm between seeds in a row) in each of the pure Bt or non-Bt maize fields, and 5400 plants for each RIB planting which included 270 non-Bt refuge plants and 5130 Bt plants. The non-Bt maize seeds were manually planted in a uniform pattern across the RIB fields right after the Bt maize seeds were planted. At each location, two non-Bt seeds were planted ∼5 cm apart and ∼2.5 cm off center of the row of Bt maize seeds and marked with wooden stakes. After about two weeks, refuge plants were thinned to one plant for each spot and tagged with colored vinyl tape. At the same time, the Bt plant that was closest to the non-Bt plant was removed to maintain the designed plant density and spacing. Irrigation, fertilization, and other management practices were used as needed to ensure optimum growth of the maize plants. Presence/absence of the Bt proteins were confirmed by testing leaf samples from each planting pattern with QuickStix Combo ELISA Kit (EnviroLogix, ME, USA). Primary ears of the three field trials were used in analyzing Bt protein expression and assessing effect of Bt protein contamination in refuge ears on survival, growth, and development of *H. zea* with three methods: in-field observation, lab assay, and field-plus-lab assay.

### Analysis of protein expression

In each trial, primary ears of 13 non-Bt maize refuge plants were randomly sampled from RIB plantings during R2–R3 stages. At the same time, 10 Bt and 10 non-Bt maize ears were also randomly collected from the pure Bt and pure non-Bt fields, respectively. For ears that were sampled from RIB and pure-Bt plantings, five kernels from the top to bottom of each ear were removed and then individually examined for expression of Cry proteins using the QuickStix Combo kit (Figure S1). For the ears collected from pure non-Bt maize field, 25 kernels were randomly sampled from each ear and pooled for analysis to validate the absence of Bt protein expression. Because the genes Cry1A.105 and Cry2Ab2 as well as Cry34Ab1 and Cry35Ab1 were linked in SmartStax, the ELISA Combo Kit identifies the individual Bt proteins as four groups: Cry1A/Cry2Ab, Cry1F, Cry3Bb and Cry34/35Ab1.

### In-field observation

Survival, growth, and development of *H. zea* on ears from the three planting patterns were first investigated using an in-field observation method with artificial insect infestation. To ensure sufficient pollinations, artificial infestations were conducted ∼7 d after the peak of pollination when plants were at R2 stage [47]. Each in-field observation consisted of six treatments, one with ears from pure non-Bt plantings, one with ears from pure Bt maize plantings, and four with ears from RIB plantings. From each RIB planting field, 40 refuge plants, and their primary ears, were first randomly selected and then six primary ears from nearby Bt plants at each sampled refuge plant were selected as shown in Figure S2. To facilitate data presentation, the seven plants selected in each location from RIB plantings were considered as four treatments: 1) RIB refuge: the refuge plant; 2) A1-Bt: the Bt plants immediately adjacent to and within the same row as the refuge plant (two plants total); 3) A3-Bt: the 3^rd^ Bt plants on both sides of the refuge plant in the same row (two plants total), and 4) B-Bt: the closest Bt plants on both sides of the refuge plant in the two adjacent rows (two plants total). At the same time, 20 plants were also randomly selected in each of the pure Bt and pure non-Bt maize fields, respectively. Before artificial infestation, naturally occurring *H. zea* larvae/eggs, if any, were removed from the ears of the selected plants and then two neonates (<24 h old) were manually placed on the top of each ear to simulate a natural field infestation. After release of the neonates, ears were covered with 17.8-cm maize ear shoot bags (Southern Exposure Seed Exchanges, Mineral, VA, USA). The open end of the ear shoot bag was attached tightly to the ear surface so that any larval movement out of the bags would be apparent based on the exit holes in the bags.

Larval survival and development were checked at 6 d after neonate release and every 3 d thereafter until larvae moved out of the ears or died. Criteria used to assign development stages (instars) of *H. zea* were based on [48]. Under field conditions, mature larvae of *H. zea* usually drop from the ears to pupate in the soil [37], leaving boring holes in the shoot bags. For data recording purposes, individuals that exited the bags in the later observations (e.g. at 12 d, 15 d and 18 d) were considered alive. Therefore, survivorship of *H. zea* for the in-field observation was calculated based on the total live larvae inside the bags and the number of exit holes in the shoot bags. A complete block design was used for the in-field observation with trial as the block factor. There were 20 to 40 ears (or 40 to 80 larvae) for each treatment replication.

### Lab assay

Because the in-field observation described above could not measure the impact of Bt contamination in refuge ears on insect survival (e.g. pupation, adult emergence) and development after mature larvae had exited from the ears and dropped into soil for pupation, a total of four lab assays were conducted using ears collected from the three field trials in 2012 and 2013. Ears used in each lab assay were selected from the trial fields with the same sampling patterns (Figure S2) as described in the in-field observation. Selected ears along with husks and shanks were brought to the laboratory and naturally infesting larvae, if any, were removed. Ears sampled from the 1^st^ field trial in 2012 were used in the first two lab assays (Lab assay-1 and Lab assay-2), while ears collected from each of the 2^nd^ and 3^rd^ field trials in 2013 were used in the 3^rd^ (Lab-array-3) and 4^th^ (Lab-array-4) lab assays, respectively. Ears for the 1^st^, 3^rd^, and 4^th^ lab assays were collected on the same days as the artificial infestations were performed for the field trials, while ears for the 2^nd^ assay were sampled 5 d after the collections for the 1^st^ lab assay.

In the lab assay, each ear was manually infested with two neonates on the top of each ear as described for the in-field observation. To maintain a suitable moisture level and keep the maize ear fresh during the test period, after insect infestation, shanks of the ears were inserted into the water-satiated Jiffy-7 peat pellets (Jiffy Greenhouse, Fulton, KY, USA). Infested ears with the peat pellets attached were then placed into 5.7 L plastic containers (one ear/container) (Sterilite Corporation, Townsend, MA, USA) with 2–3 pieces of paper towel underneath. The insect assay containers were placed into growth chambers maintained at 28°C, ∼50% RH, 16L: 8D photoperiod. Survival, growth, and development of *H. zea* were checked after 6 d and every 3 d thereafter until adult emergence or death. A complete block (growth chamber) design was used in each lab assay with 3 (Lab-array-1) or 4 (Lab-array-2, -3 and -4) replications and 8–10 ears/replication.

### Field-plus-lab assay

A total of three field-plus-lab assays were conducted using ears collected from the two field trials in 2013; two assays using ears from the field trial in Franklin Parish and another from the trial in Rapides Parish. Because there were virtually no live larvae on the ears of Bt maize plants when ear samplings were performed, each assay consisted of only two treatments: 1) refuge ears from RIB and 2) ears of pure non-Bt maize planting. In each assay, ears with naturally occurring larvae along with husks and shanks were sampled from the field at the peak population of the 3^rd^ instar stages and brought to the laboratory. The initial number of larvae and their corresponding developmental stages on each ear were recorded while leaving larvae in place inside the ears. The ears with the peat pellets attached as described above, along with intact naturally occurring larvae, were then placed into plastic containers and maintained in the same conditions as described in the lab assay. Survival, growth, and development of insects were checked every 2–3 days until adult emergence or death. A complete block design was used in the field-plus-lab assay with assay as the block factor. The number of ears used in each treatment replication varied from 20 to 100 depending on the number of infested ears available.

### Data analysis

Percent of kernels containing one or more Bt proteins was calculated based on the number of kernels expressing the Bt proteins divided by the total kernels assayed. Based on presence/absence of the protein expression in kernels of the refuge ears, χ^2^-tests were used to determine if the four gene groups in SmartStax segregated independently. The χ^2^ value was determined using the equation: χ^2^ = (n/100) [(O−E)^2^/E+(E−O)^2^/(100−E)]. Here, n = number of kernels examined, O = observed percentage of kernels expressing the Bt proteins, and E = expected percentage of kernels expressing the Bt proteins. The E value for a combination of two or more proteins was based on the assumption of independent segregation. For example, expected frequency of Cry1A/Cry2A+Cry3B was calculated using the observed frequency of Cry1A/Cry2A multiplied by the observed frequency of Cry3B.

Insect developmental stages were converted to a development index: 1 = 1^st^ instar, 2 = 2^nd^ instar, …, 6 = 6^th^ instar, 7 = pupa as described in Yang *et al*. [6]. Data on insect survivorship, pupation, and moth emergence rate were transformed to arcsine square-root value, while number of insect, development index, and pupal mass were converted to ln (x+1) scale for normal distributions [49]. Transformed data were then analysed using one-way analysis of variance (ANOVA) [50]. In addition, data for each variable observed in the lab assay were also pooled across the four assays and the pooled data were analysed using mixed models with assay as a random factor [50]. For all ANOVAs, treatment means were compared and separated by Tukey’s HSD tests at α = 0.05 level. Untransformed data are presented in the tables and figures.

## Supporting Information

Figure S1 Demonstration of Bt protein expression in individual kernels removed from ears of pure SmartStax planting (A) and refuge ears of RIB (B) on QuickStix Combo ELISA test strips (EnviroLogix, ME, USA).
(TIF)

Figure S2 A diagram showing the seven plants (four treatments) in each randomly selected location in a RIB planting that was used for the in-field observations and lab-bioassays. RIB refuge: the refuge plant; A1-Bt: the Bt plants immediately adjacent and within the same row as the refuge plant; A3-Bt: the 3^rd^ Bt plants on both sides of the refuge plant in the same row; and B-Bt: the closest Bt plants on both sides of the refuge plant in the two adjacent rows.
(TIF)

Table S1 Percentage (mean ± sem) of individual kernels expressing Bt proteins in SmartStax maize in pure Bt, pure non-Bt, and RIB plantings.
(DOCX)

Table S2 In-field observation of survivorship and development (mean ± sem) of *H. zea* on ears of SmartStax Bt and non-Bt maize plants in three planting patterns.
(DOCX)

Table S3 Lab assay on survivorship (mean ± sem) of *H. zea* on ears of SmartStax Bt and non-Bt maize plants in three planting patterns.
(DOCX)

Table S4 Lab assay on development index, pupal weight, and development time (mean ± sem) of *H. zea* on ears of SmartStax Bt and non-Bt maize plants in three planting patterns.
(DOCX)

Table S5 Field-plus-lab assay on pupal mass (mean ± sem) of *H. zea* on ears of RIB refuge and pure non-Bt plants.
(DOCX)

## References

[1] James C (2013) Global status of commercialized biotech/GM crops: 2013. ISAAA Brief No. 45. ISAAA: Ithaca, NY, USA.

[2] NASS (National Agricultural Statistics Service) (2013). Acreage. USDA, Washington DC. USA. http://usda01.library.cornell.edu/usda/current/Acre/Acre-06-28-2013.pdf.

[3] Siebert MW, Nolting SP, Hendrix W, Dhavala S, Craig C, et al (2012) Evaluation of corn hybrids expressing Cry1F, Cry1A.105, Cry2Ab2, Cry34Ab1/Cry35Ab1, and Cry3Bb1 against southern United States insect pests. J Econ Entomol 105: 1825–1834. 23156183 10.1603/ec12155

[4] Hutchison WD, Burkness EC, Mitchell PD, Moon RD, Leslie TW, et al (2010) Areawide suppression of European corn borer with Bt maize reaps savings to non-Bt maize growers. Science 330: 222–225. 20929774 10.1126/science.1190242

[5] Edgerton MD, Fridgen J, Anderson-Jr JR, Ahlgrim J, Criswell M, et al (2012) Transgenic insect resistance traits increase corn yield and yield stability. Nat Biotechnol 30: 493–496. 22678382 10.1038/nbt.2259

[6] Yang F, Kerns DL, Head GP, Leonard BR, Niu Y, et al (2014) Occurrence, distribution, and ear damage of *Helicoverpa zea* (Lepidoptera: Noctuidae) in mixed plantings of non-Bt and Bt corn containing SmartStax traits. Crop Prot 55: 127–132.

[7] Niu Y, Yang F, Dangal V, Huang F (2014) Larval survival and plant injury of Cry1F-susceptible, -resistant, and -heterozygous fall armyworm (Lepidoptera: Noctuidae) on non-Bt and Bt corn containing single or pyramided genes. Crop Prot 59: 22–28.

[8] Gould F (1998) Sustainability of transgenic insecticidal cultivars: integrating pest genetics and ecology. Ann Rev Entomol 43: 701–726. 15012402 10.1146/annurev.ento.43.1.701

[9] Van Rensburg JBJ (2007) First report of field resistance by the stem borer, *Busseola fusca* (Fuller) to Bt-transgenic maize. S Afr J Plant Soil 24: 147–151.

[10] Storer NP, Babcock JM, Schlenz M, Meade T, Thompson GD, et al (2010) Discovery and characterization of field resistance to Bt maize: *Spodoptera frugiperda* (Lepidoptera: Noctuidae) in Puerto Rico. J Econ Entomol 103: 1031–1038. 20857709 10.1603/ec10040

[11] Dhurua S, Gujar GT (2011) Field-evolved resistance to Bt protein Cry1Ac in the pink bollworm, *Pectinophora gossypiella* (Saunders) (Lepidoptera: Gelechiidae) from India. Pest Manag Sci 67: 898–903. 21438121 10.1002/ps.2127

[12] Gassmann AJ, Petzold-Maxwell JL, Keweshan RS, Dunbar MW (2011) Field-evolved resistance to Bt maize by western corn rootworm. PloS ONE 6: e22629 doi:10.1371/journal.pone.0022629 21829470 10.1371/journal.pone.0022629 PMC3146474

[13] Huang F, Andow DA, Buschman LL (2011) Success of the high dose/refuge resistance management strategy after 15 years of Bt crop use in North America. Entomol Exp App 140: 1–16.

[14] Tabashnik BE, Brévault T, Carrière Y (2013) Insect resistance to Bt crops: lessons from the first billion acres. Nat Biotechnol 31: 510–521. 23752438 10.1038/nbt.2597

[15] Matten SR, Frederick RJ, Reynolds AH (2012) United States Environmental Protection Agency insect resistance management programs for plant-incorporated protectants and use of simulation modeling. Regulation of agricultural biotechnology: The United States and Canada, eds Wozniak CA, McHughen A (Springer), 175−267.

[16] Ostlie KR, Hutchison WD, Hellmich RL (1997) Bt corn & European corn borer: long term success through resistance management. North Central Regional Extension Publication NCR 602.

[17] U.S. Environmental Protection Agency (2001). Biopesticide registration action document: *Bacillus thuringiensis* plant-incorporated protectants. http://www.epa.gov/oppbppd1/biopesticides/pips/bt_brad2/1-overview.pdf. Accessed 17 July 2014.

[18] Mallet J, Porter P (1992) Preventing insect adaptation to insect-resistant crops: are seed mixtures or refugia the best strategy? Proc R Soc Lond B 250: 165–169.

[19] U.S. Environmental Protection Agency (2010) Terms and conditions for Bt corn registrations 30 Sept 2010. Office of Pesticide Programs, Washington, DC. http://www.epa.gov/oppbppd1/biopesticides/pips/bt-corn-terms-conditions.pdf. Accessed 17 July 2014.

[20] Onstad DW, Mitchell PD, Hurley TM, Lundgren JG, Porter RP, et al (2011) Seeds of change: corn seed mixtures for resistance management and IPM. J Econ Entomol 104: 343–352. 21510178 10.1603/ec10388

[21] Roush RT (1998) Two-toxin strategies for management of insecticidal transgenic crops: can pyramiding succeed where pesticide mixtures have not? Phil Trans R Soc Lond B 353: 1777–1786.

[22] Zhao JZ, Cao J, Li Y, Collins HL, Roush RT, et al (2003) Transgenic plants expressing two *Bacillus thuringiensis* toxins delay insect resistance evolution. Nat Biotechnol 21: 1493–1497. 14608363 10.1038/nbt907

[23] Difonzo C, Collen E (2012) Handy Bt trait table. Available at: http://www3.ag.purdue.edu/agry/PCPP/Documents/Entry%20forms/Handy_Bt_Trait_Table.pdf.

[24] Alyokhin A (2011) Scant evidence supports EPA’s pyramided Bt corn refuge size of 5%. Nat Biotech 29: 577–578. 10.1038/nbt.1911 21747379

[25] Kang J, Onstad DW, Hellmich RL, Moser SE, Hutchison WD, et al (2012) Modeling the impact of cross-pollination and low toxin expression in corn kernels on adaptation of European corn borer (Lepidoptera: Crambidae) to transgenic insecticidal corn. Environ Entomol 41: 200–211. 22649850 10.1603/en11133

[26] U.S. Environmental Protection Agency (2010). Biopesticides registration action document: *Bacillus thuringiensis* Cry1A.105 and Cry2Ab2 insecticidal proteins and the genetic material necessary for their production in corn. http://www.epa.gov/oppbppd1/biopesticides/pips/mon-89034-brad.pdf. Accessed 17 July 2014.

[27] Chilcutt CF, Tabashnik BE (2004) Contamination of refuges by *Bacillus thuringiensis* toxin genes from transgenic maize. Proc Natl Acad Sci USA 101: 7526–7529. 15136739 10.1073/pnas.0400546101 PMC419639

[28] Burkness EC, O’Rourke PK, Hutchison WD (2011) Cross-pollination of nontransgenic corn ears with transgenic Bt corn: efficacy against lepidopteran pests and implications for resistance management. J Econ Entomol 104: 1476–1479. 22066174 10.1603/ec11081

[29] Burkness EC, Hutchison WD (2012) Bt pollen dispersal and Bt kernel mosaics: integrity of non-Bt refugia for lepidopteran resistance management in maize. J Econ Entomol 105: 1477–1870. 23156176 10.1603/ec12128

[30] Davis PM, Onstad DW (2000) Seed mixtures as a resistance management strategy for European corn borers (Lepidoptera: Crambidae) infesting transgenic corn expressing Cry1Ab protein. J Econ Entomol 93: 937–948. 10902353 10.1603/0022-0493-93.3.937

[31] Carroll MW, Head G, Caprio M (2012) When and where a seed mix refuge makes sense for managing insect resistance to Bt plants. Crop Prot 38: 74–79.

[32] Carroll MW, Head G, Caprio M, Stork L (2013) Theoretical and empirical assessment of a seed mix refuge in corn for southwestern corn borer. Crop Prot 49: 58–65.

[33] Lindgren PD, Westbrook JK, Bryant VM, Raulston JR, Esquivel JF, et al (1994) Origin of corn earworm (Lepidoptera: Noctuidae) migrants as determined by citrus pollen markers and synoptic weather systems. Environ Entomol 23: 562–570.

[34] Heuberger S, Ellers-Kirk C, Yafuso C, Gassmann AJ, Tabashnik BE, et al (2008) Effects of refuge contamination by transgenes on Bt resistance in pink bollworm (Lepidoptera: Gelechiidae). J Econ Entomol 101: 504–514. 18459418 10.1603/0022-0493(2008)101[504:eorcbt]2.0.co;2

[35] Chilcutt CF, Odvody GN, Correa JC, Remmers J (2007) Effects of *Bacillus thuringiensis* transgenic corn on corn earworm and fall armyworm (Lepidoptera: Noctuidae) densities. J Econ Entomol 100: 327–334. 17461054 10.1603/0022-0493(2007)100[327:eobttc]2.0.co;2

[36] Burkness EC, Dively G, Patton T, Morey AC, Hutchison WD (2010) Novel Vip3A *Bacillus thuringiensis* (Bt) maize approaches high-dose efficacy against *Helicoverpa zea* (Lepidoptera: Noctuidae) under field conditions. GM crops 1: 1–7. 21844691 10.4161/gmcr.1.5.14765

[37] Capinera JL (2000) Corn earworm, Helicoverpa ( = Heliothis) zea (Boddie) (Lepidoptera: Noctuidae). Florida Cooperative Extension Service, Institute of Food and Agricultural Sciences, University of Florida. EENY-145 (IN302).

[38] Kranthi KR (2006) On the variability of Cry1Ac expression in commercialized *Bt* cotton varieties in India. Curr Sci 90: 1170–1172.

[39] Huang F, LeonardBR, Andow DA (2007) F_2_ screen for resistance to a *Bacillus thuringiensis*-maize hybrid in the sugarcane borer (Lepidoptera: Crambidae). Bull Entomol Res 97: 1–8. 17916262 10.1017/S000748530700510X

[40] Poongothai S, Ilavarasan R, Karrunakaran CM (2013) Cry 1Ac levels and biochemical variations in Bt cotton as influenced by tissue maturity and senescence. Int J Biol Res 1: 49–55.

[41] Leahy TC, Andow DA (1994) Egg weight, fecundity, and longevity are increased by adult feeding in *Ostrinia nubilalis* (Lepidoptera: Pyralidae). Ann Entomol Soc Am 87: 342–349.

[42] Goldstein JA, Mason CE, Pesek J (2010) Dispersal and movement behavior of neonate European corn borer (Lepidoptera: Crambidae) on non-Bt and transgenic Bt corn. J Econ Entomol 103: 331–339. 20429445 10.1603/ec09304

[43] Ives AR, Glaum PR, Ziebarth NL, Andow DA (2011) The evolution of resistance to two-toxin pyramided transgenic crops. Ecol Appl 21: 503–515. 21563580 10.1890/09-1869.1

[44] Razze JM, Mason CE (2012) Dispersal behavior of neonate European corn borer (Lepidoptera: Crambidae) on Bt corn. J Econ Entomol 105: 1214–23. 22928300 10.1603/ec11288

[45] Wangila DS, Leonard BR, Ghimire MN, Bai Y, Zhang L, et al (2013) Occurrence and larval movement of *Diatraea saccharalis* (Lepidoptera: Crambidae) in seed mixes of non-Bt and Bt pyramid corn. Pest Manag Sci 69: 1163–1172. 23456950 10.1002/ps.3484

[46] Bannert M (2006) Simulation of transgenic pollen dispersal by use of different grain colour maize. Ph.D. Dissertation, Swiss Federal Institute of Technology Zurich. Available at: http://www.agrisite.de/doc/ge_img/pollen-swiss.pdf.

[47] Abendroth LJ, Elmore RW, Boyer MJ, Marlay SK (2011) Corn growth and development. Ames, IA: *PMR* 1009, Iowa State University Extension, IA, US.

[48] Capinera JL (2001) Handbook of vegetable pests. Academic Press, San Diego, California, USA. 396–397.

[49] Zar JH (1984) *Biostatistical analysis, 2nd ed*. Prentice-Hall, Englewood Cliffs, NJ, USA.

[50] SAS Institute (2010) *SAS/STAT User*’*s Third Edition*, SAS Institute Inc, Cary, NC, USA.

