# Supplementary figures and images for: A Challenge for the Seed Mixture Refuge Strategy in Bt Maize: Impact of Cross-Pollination on an Ear-Feeding Pest, Corn Earworm
Source: PLoS One. 2014 Nov 19;9(11):e112962. doi: 10.1371/journal.pone.0112962 (PMC4237366; doi:10.1371/journal.pone.0112962)

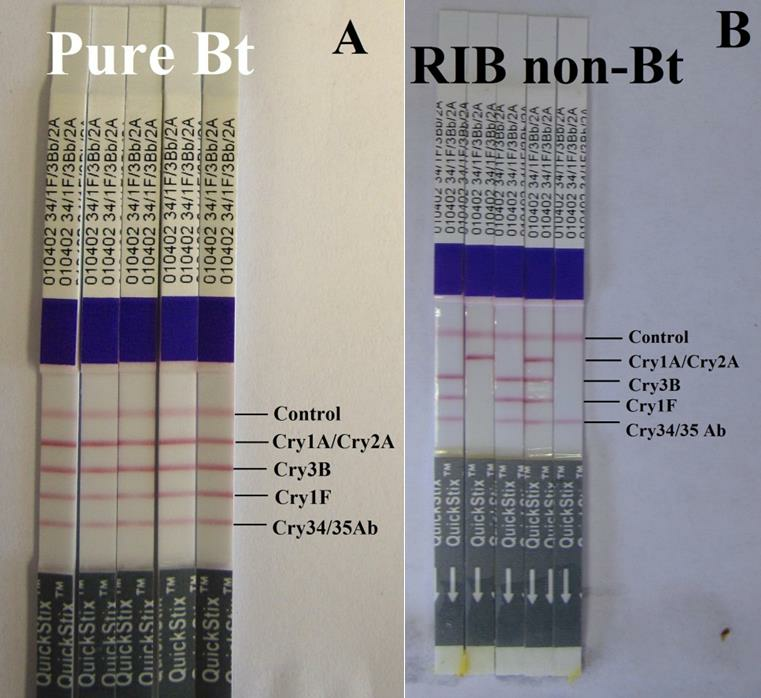

Supplement: Figure S1 — Demonstration of Bt protein expression in individual kernels removed from ears of pure SmartStax planting (A) and refuge ears of RIB (B) on QuickStix Combo ELISA test strips (EnviroLogix, ME, USA). (TIF) [file pone.0112962.s001.tif]

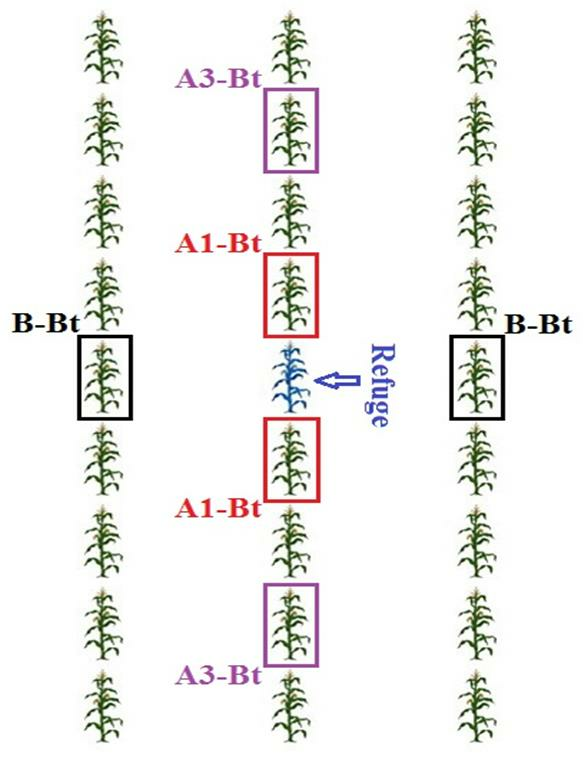

Supplement: Figure S2 — A diagram showing the seven plants (four treatments) in each randomly selected location in a RIB planting that was used for the in-field observations and lab-bioassays. RIB refuge: the refuge plant; A1-Bt: the Bt plants immediately adjacent and within the same row as the refuge plant; A3-Bt: the 3rd Bt plants on both sides of the refuge plant in the same row; and B-Bt: the closest Bt plants on both sides of the refuge plant in the two adjacent rows. (TIF) [file pone.0112962.s002.tif]
