# Supplementary material for: A Challenge for the Seed Mixture Refuge Strategy in Bt Maize: Impact of Cross-Pollination on an Ear-Feeding Pest, Corn Earworm
Source: PLoS One. 2014 Nov 19;9(11):e112962. doi: 10.1371/journal.pone.0112962 (PMC4237366; doi:10.1371/journal.pone.0112962)
Supplement: Table S1 — Percentage (mean ± sem) of individual kernels expressing Bt proteins in SmartStax maize in pure Bt, pure non-Bt, and RIB plantings. (DOCX) [file pone.0112962.s003.docx]

**Table S1.** Percentage (mean ± sem) of individual kernels expressing Bt proteins in SmartStax maize in pure Bt, pure non-Bt, and RIB plantings ^a^.

| **Bt protein group** | **Field trial 1** | | | | | **Field trial 2** | | | | | **Field trial 3** | | | | | **Pooled^c^** | | | | |
| --- | --- | --- | --- | --- | --- | --- | --- | --- | --- | --- | --- | --- | --- | --- | --- | --- | --- | --- | --- | --- |
|  | **Pure Bt (%)** | **Pure non-Bt (%)** | **RIB refuge** | | | **Pure Bt (%)** | **Pure non-Bt (%)** | **RIB refuge** | | | **Pure Bt (%)** | **Pure non-Bt (%)** | **RIB refuge** | | | **Pure Bt (%)** | **Pure non-Bt (%)** | **RIB refuge** | | |
|  |  |  | **O^b^ (%)** | **E^b^ (%)** | **χ^2^** |  |  | **O (%)** | **E (%)** | **χ^2^** |  |  | **O (%)** | **E (%)** | **χ^2^** |  |  | **O (%)** | **E (%)** | **χ^2^** |
| Cry1A/Cry2A | 100 | 0 | 47.7 ± 8.0 | --- | --- | 100 | 0 | 50.8 ± 7.0 | --- | --- | 100 | 0 | 49.2 ± 7.7 | --- | --- | 100 | 0 | 49.2 ± 4.3 | --- | --- |
| Cry3B | 100 | 0 | 53.9 ± 4.7 | --- | --- | 100 | 0 | 96.9 ± 3.1 | --- | --- | 100 | 0 | 72.3 ± 5.8 | --- | --- | 100 | 0 | 74.4 ± 3.9 | --- | --- |
| Cry1F | 100 | 0 | 56.9 ± 5.9 | --- | --- | 100 | 0 | 41.5 ± 5.3 | --- | --- | 100 | 0 | 36.9 ± 6.7 | --- | --- | 100 | 0 | 45.1 ± 3.7 | --- | --- |
| Cry34/35Ab | 100 | 0 | 46.2 ± 6.6 | --- | --- | 100 | 0 | 60.0 ± 4.5 | --- | --- | 100 | 0 | 49.2 ± 7.7 | --- | --- | 100 | 0 | 51.8 ± 3.7 | --- | --- |
| Cry1A/Cry2A + Cry3B | 100 | 0 | 26.2 ± 5.7 | 25.7 | 0.01 | 100 | 0 | 50.8 ± 7.0 | 49.2 | 0.06 | 100 | 0 | 36.9 ± 7.5 | 35.6 | 0.05 | 100 | 0 | 38.0 ± 4.1 | 36.6 | 0.15 |
| Cry1A/Cry2A + Cry1F | 100 | 0 | 26.2 ± 6.9 | 27.2 | 0.04 | 100 | 0 | 26.2 ± 5.3 | 21.1 | 1.00 | 100 | 0 | 13.9 ± 4.7 | 18.2 | 0.83 | 100 | 0 | 22.1 ± 3.4 | 22.2 | 0.00 |
| Cry1A/Cry2A + Cry34/35Ab | 100 | 0 | 12.3 ± 3.6 | 22.0 | 3.56 | 100 | 0 | 23.8 ± 6.3 | 30.5 | 1.69 | 100 | 0 | 3.1 ± 2.1 | 24.2 | 15.81^s^ | 100 | 0 | 12.8 ± 2.8 | 25.5 | 16.50^s^ |
| Cry3B + Cry1F | 100 | 0 | 33.9 ± 5.3 | 30.7 | 0.30 | 100 | 0 | 41.5 ± 5.3 | 40.3 | 0.04 | 100 | 0 | 33.9 ± 5.7 | 26.7 | 1.70 | 100 | 0 | 36.4 ± 3.1 | 33.6 | 0.69 |
| Cry3B + Cry34/35Ab | 100 | 0 | 29.2 ± 7.4 | 24.9 | 0.65 | 100 | 0 | 60.0 ± 4.5 | 58.2 | 0.09 | 100 | 0 | 36.9 ± 5.9 | 35.6 | 0.05 | 100 | 0 | 42.1 ± 4.0 | 38.5 | 1.04 |
| Cry1F + Cry34/35Ab | 100 | 0 | 30.8 ± 4.9 | 26.3 | 0.67 | 100 | 0 | 21.5 ± 4.8 | 24.9 | 0.39 | 100 | 0 | 24.6 ± 5.1 | 18.2 | 1.80 | 100 | 0 | 25.6 ± 2.8 | 23.4 | 0.55 |
| Cry1A/Cry2A + Cry3B + Cry1F | 100 | 0 | 13.9 ± 4.2 | 14.6 | 0.03 | 100 | 0 | 26.2 ± 5.3 | 20.4 | 1.33 | 100 | 0 | 12.3 ± 4.3 | 13.1 | 0.04 | 100 | 0 | 17.4 ± 2.8 | 16.5 | 0.12 |
| Cry1A/Cry2A + Cry3B + Cry34/35Ab | 100 | 0 | 6.2 ± 2.7 | 11.9 | 2.05 | 100 | 0 | 23.1 ± 6.3 | 29.5 | 1.29 | 100 | 0 | 1.5 ± 1.5 | 17.5 | 11.47^s^ | 100 | 0 | 10.3 ± 2.7 | 19.0 | 9.69^s^ |
| Cry1A/Cry2A + Cry1F + Cry34/35Ab | 100 | 0 | 7.7 ± 3.6 | 12.5 | 1.37 | 100 | 0 | 9.2 ± 4.3 | 12.7 | 0.71 | 100 | 0 | 1.5 ± 1.5 | 9.0 | 4.42^s^ | 100 | 0 | 6.2 ± 2.0 | 11.5 | 5.48^s^ |
| Cry3B + Cry1F + Cry34/35Ab | 100 | 0 | 21.5 ± 6.2 | 14.2 | 2.87 | 100 | 0 | 21.5 ± 4.8 | 24.2 | 0.25 | 100 | 0 | 23. 1± 5.0 | 13.1 | 5.68^s^ | 100 | 0 | 22.1 ± 3.0 | 17.4 | 2.94 |
| Cry1A/Cry2A + Cry3B + Cry1F + Cry34/35Ab | 100 | 0 | 4.6 ± 2.4 | 6.8 | 0.49 | 100 | 0 | 9.2 ± 4.3 | 12.3 | 0.57 | 100 | 0 | 1.5 ± 1.5 | 6.5 | 2.63 | 100 | 0 | 5.1 ± 1.8 | 8.6 | 2.99 |
| Negative | --- | 100 | 9.2 ± 3.7 | --- | --- | --- | 100 | 3.1 ± 3.1 | --- | --- | --- | 100 | 4.6 ± 3.3 | --- | --- | --- | 100 | 5.6 ± 1.9 | --- | --- |

^a^ In each field trial, 5 individual kernels per ear with 10 ears (n = 50) were examined for ears of pure Bt maize plantings; 25 kernels per ear with 10 ears (n = 250) were tested for ears of pure non-Bt maize plantings; and for refuge ears in RIB, 5 individual kernels per ear with 13 ears (n = 65 for each trial and n = 195 for the pooled data) were assayed. Pure Bt: primary ears of pure Bt maize planting; pure non-Bt: primary ears of pure non-Bt maize planting; and RIB refuge: primary ears of the refuge plants in the RIB planting.

^b^ O: observation frequency; and E: expected frequency based on the assumption of independent segregation. For example, expected frequency of Cry1A/Cry2A + Cry3B was calculated using the observed frequency of Cry1A/Cry2A multiplied by the observed frequency of Cry3B. Then χ^2^ was determined using the equation: χ^2^ = (n/100) [(O-E)^2^/E + (E-O)^2^/(100-E)].

^c^ Pooled data across the three trials.

^s^ Indicates significantly different from the assumption of independent segregation in χ^2^-tests with df =1 at the α = 0.05 level.
