# Supplementary material for: A Challenge for the Seed Mixture Refuge Strategy in Bt Maize: Impact of Cross-Pollination on an Ear-Feeding Pest, Corn Earworm
Source: PLoS One. 2014 Nov 19;9(11):e112962. doi: 10.1371/journal.pone.0112962 (PMC4237366; doi:10.1371/journal.pone.0112962)
Supplement: Table S2 — In-field observation of survivorship and development (mean ± sem) of H. zea on ears of SmartStax Bt and non-Bt maize plants in three planting patterns. (DOCX) [file pone.0112962.s004.docx]

**Table S2.** In-field observation of survivorship and development (mean ± sem) of *H. zea* on ears of SmartStax Bt and non-Bt maize plants in three planting patterns ^a^.

| Ears | | Survivorship (%)^b^ | | | | | Development index^c^ | | |
| --- | --- | --- | --- | --- | --- | --- | --- | --- | --- |
|  |  | 6-d | 9-d | 12-d | 15-d | 18-d | 6-d | 9-d | 12-d |
| Pure Bt | | 1.68 ± 0.82 a  5.42 ± 2.92 a | 0.00 ± 0.00 a  0.83 ± 0.42 a | 0.00 ± 0.00 a  0.42 ± 0.42 a | 0.00 ± 0.00 a  0.00 ± 0.00 a | 0.00 ± 0.00 a  0.00 ± 0.00 a | 2.50 ± 0.50 a  2.35 ± 0.18 a | ---  3.50 ± 0.50 b | ---  3.00 a |
| RIB | A1-Bt |  |  |  |  |  |  |  |  |
|  | A3-Bt | 5.42 ± 0.83 a | 0.83 ± 0.42 a | 0.00 ± 0.00 a | 0.00 ± 0.00 a | 0.00 ± 0.00 a | 2.47 ± 0.15 a | 2.50 ± 0.50 a | --- |
|  | B-Bt | 5.00 ± 1.91 a | 1.25 ± 1.25 a | 0.00 ± 0.00 a | 0.00 ± 0.00 a | 0.00 ± 0.00 a | 2.44 ± 0.29 a | 3.00 ab | --- |
|  | Refuge | 62.27 ± 3.97 b  61.20 ± 3.61 b | 38.67 ± 1.88 b  53.77 ± 5.65 c | 24.77 ± 3.84 b  45.54 ± 6.56 c | 19.12 ± 1.77 b  43.87 ± 2.17 c | 16.16 ± 2.08 b  43.87 ± 2.17 c | 2.81 ± 0.01 a  3.78 ± 0.17 b | 3.90 ± 0.07 b  5.40 ± 0.15 c | 4.91 ± 0.29 b  6.36 ± 0.05 c |
| Pure non-Bt | |  |  |  |  |  |  |  |  |
| F-test | F-value | *F*_5, 10_= 96.69 | *F*_5, 10_= 74.31 | *F*_5, 10_= 133.63 | *F*_5, 10_= 606.47 | *F*_5, 10_= 501.23 | *F*_5, 9_= 6.14 | *F*_4, 4_= 20.26 | *F*_2, 2_= 37.92 |
|  | P-value | < 0.0001 | < 0.0001 | < 0.0001 | < 0.0001 | < 0.0001 | 0.01 | 0.006 | 0.026 |

^a^ Pure Bt: pure Bt maize planting; pure non-Bt: pure non-Bt maize planting; RIB refuge: the refuge plants in the RIB planting; A1-Bt: the Bt plants immediately adjacent and within the same row as the refuge plant in RIB planting; A3-Bt: the 3^rd^ Bt plants on both sides of the refuge plant in the same row in RIB planting, and B-Bt: the closest Bt plants on both sides of the refuge plant in the two adjacent rows in RIB planting. Means were calculated based on three independent field trials (treated as a random factor). Sample size for each mean for measuring survivorship was 240 larvae for RIB and 120 larvae for pure Bt and pure non-Bt. Sample size for determining larval development on pure non-Bt and RIB refuge was 55-150 larvae and on Bt plants was 1-13 larvae. Means in a column followed by a different letter were significantly different (Tukey’s HSD test, α = 0.05). Pure Bt: pure Bt maize planting; pure non-Bt: pure non-Bt maize planting; RIB refuge: the refuge plants in the RIB planting.

^b^ Insect survivorship after 12 d was estimated based on the sum of the number of live larvae inside the ears and the holes bored by late instar larvae in the shoot bags.

^c^ Insect development were converted to a development index: 1= 1^st^ instar, 2= 2^nd^ instar, …, 6= 6^th^ instar, 7= pupal stage.
