# Supplementary material for: A Challenge for the Seed Mixture Refuge Strategy in Bt Maize: Impact of Cross-Pollination on an Ear-Feeding Pest, Corn Earworm
Source: PLoS One. 2014 Nov 19;9(11):e112962. doi: 10.1371/journal.pone.0112962 (PMC4237366; doi:10.1371/journal.pone.0112962)
Supplement: Table S3 — Lab assay on survivorship (mean ± sem) of H. zea on ears of SmartStax Bt and non-Bt maize plants in three planting patterns. (DOCX) [file pone.0112962.s005.docx]

**Table S3.** Lab assay on survivorship (mean ± sem) of *H. zea* on ears of SmartStax Bt and non-Bt maize plants in three planting patterns ^a^.

| Assay^b^ | Ears | | | | Survivorship (%) | | | | | | | | | | |  |
| --- | --- | --- | --- | --- | --- | --- | --- | --- | --- | --- | --- | --- | --- | --- | --- | --- |
|  |  |  |  |  | 6-d | 9-d | 12-d | 15-d | | 18-d | | NTP^c^ | | NTA^d^ | |  |
| Lab assay-1 | Pure Bt | | | | 6.67 ± 4.41 a  1.67 ± 1.67 a | 0.00 ± 0.00 a  0.00 ± 0.00 a | 0.00 ± 0.00 a  0.00 ± 0.00 a | 0.00 ± 0.00 a  0.00 ± 0.00 a | | 0.00 ± 0.00 a  0.00 ± 0.00 a | | 0.00 ± 0.00 a  0.00 ± 0.00 a | | 0.00 ± 0.00 a  0.00 ± 0.00 a | |  |
|  | RIB | A1-Bt | | |  |  |  |  |  |  |  |  |  |  |  |  |
|  |  | A3-Bt | | | 1.67 ± 1.67 a | 0.00 ± 0.00 a | 0.00 ± 0.00 a | 0.00 ± 0.00 a | | 0.00 ± 0.00 a | | 0.00 ± 0.00 a | | 0.00 ± 0.00 a | |  |
|  |  | B-Bt | | | 3.33 ± 3.33 a | 0.00 ± 0.00 a | 0.00 ± 0.00 a | 0.00 ± 0.00 a | | 0.00 ± 0.00 a | | 0.00 ± 0.00 a | | 0.00 ± 0.00 a | |  |
|  |  | Refuge | | | 73.67 ± 11.29 b | 53.50 ± 8.54 b | 41.70 ± 5.50 b | 27.77 ± 2.77 b | | 22.93 ± 2.07 b | | 9.73 ± 5.02 b | | 4.17 ± 4.17 a | |  |
| Pure non-Bt | | | | 80.33 ± 12.25 b | 64.83 ± 6.67 b | 55.67 ± 3.20 c | 44.40 ± 3.23 c | | 40.73 ± 4.91 c | | 40.73 ± 4.91 c | | 33.30 ± 6.41 b | |  |  |
| F-test | | | F-value | *F*_5, 10_= 19.22 | *F*_5, 10_= 98.19 | *F*_5, 10_= 227.65 | *F*_5, 10_= 339.47 | | *F*_5, 10_= 194.05 | | *F*_5, 10_= 23.94 | | *F*_5, 10_= 18.73 | |  |  |
|  |  | | | P-value | < 0.0001 | < 0.0001 | < 0.0001 | < 0.0001 | | < 0.0001 | | < 0.0001 | | < 0.0001 | |  |
| Lab assay-2 | Pure Bt | | | | 1.25 ± 1.25 a | 0.00 ± 0.00 a | 0.00 ± 0.00 a | 0.00 ± 0.00 a | | 0.00 ± 0.00 a | | 0.00 ± 0.00 a | | 0.00 ± 0.00 a | |  |
|  | RIB | A1-Bt | | | 4.06 ± 1.39 a | 0.00 ± 0.00 a | 0.00 ± 0.00 a | | 0.00 ± 0.00 a | | 0.00 ± 0.00 a | | 0.00 ± 0.00 a | | 0.00 ± 0.00 a | |
|  |  | A3-Bt | | | 1.25 ± 1.25 a | 0.00 ± 0.00 a | 0.00 ± 0.00 a | 0.00 ± 0.00 a | | 0.00 ± 0.00 a | | 0.00 ± 0.00 a | | 0.00 ± 0.00 a | |  |
|  |  | B-Bt | | | 5.94 ± 2.57 a | 1.56 ± 1.56 a  60.63 ± 5.43 b | 1.56 ± 1.56 a | 1.56 ± 1.56 a | | 1.56 ± 1.56 a | | 0.00 ± 0.00 a | | 0.00 ± 0.00 a | |  |
|  |  | Refuge | | | 80.63 ± 8.17 b |  | 47.50 ± 5.68 b | 36.25 ± 3.35 b | | 25.63 ± 8.61 b | | 6.56 ± 4.72 b | | 6.56 ± 4.72 b | |  |
| Pure non-Bt | | | | 82.50 ± 3.06 b | 66.88 ± 1.08 b | 60.63 ± 4.80 c | 48.44 ± 7.13 c | | 45.31 ± 5.60 c | | 45.31 ± 5.60 c | | 40.00 ± 6.63 c | |  |  |
| F-test | | | F-value | *F*_5, 15_= 58.85  < 0.0001 | *F*_5, 15_= 194.83  < 0.0001 | *F*_5, 15_= 110.41  < 0.0001 | *F*_5, 15_= 76.03  < 0.0001 | | *F*_5, 15_= 32.19  < 0.0001 | | *F*_5, 15_= 33.01  < 0.0001 | | *F*_5, 15_= 25.95  < 0.0001 | |  |  |
|  |  |  | P-value |  |  |  |  |  |  |  |  |  |  |  |  |  |
| Lab assay-3 | Pure Bt | | | | 2.50 ± 1.44 a | 1.25 ± 1.25 a | 0.00 ± 0.00 a | 0.00 ± 0.00 a | | 0.00 ± 0.00 a | | 0.00 ± 0.00 a | | 0.00 ± 0.00 a | |  |
|  | RIB | A1-Bt | | | 7.50 ± 3.23 a | 2.50 ± 1.44 a | 1.25 ± 1.25 a | 1.25 ± 1.25 a | | 0.00 ± 0.00 a | | 0.00 ± 0.00 a | | 0.00 ± 0.00 a | |  |
|  |  | A3-Bt | | | 3.75 ± 1.25 a | 1.25 ± 1.25 a | 1.25 ± 1.25 a | 0.00 ± 0.00 a | | 0.00 ± 0.00 a | | 0.00 ± 0.00 a | | 0.00 ± 0.00 a | |  |
|  |  | B-Bt | | | 7.50 ± 1.44 a | 1.25 ± 1.25 a 57.50 ± 3.22 b | 0.00 ± 0.00 a | 0.00 ± 0.00 a | | 0.00 ± 0.00 a | | 0.00 ± 0.00 a | | 0.00 ± 0.00 a | |  |
|  |  | Refuge | | | 81.25 ± 3.75 b |  | 38.75 ± 3.75 b | 25.00 ± 4.08 b | | 11.25 ± 3.15 b | | 5.00 ± 2.04 b | | 3.75 ± 1.25 b | |  |
| Pure non-Bt | | | | 81.25 ± 3.15 b | 62.50 ± 1.44 b | 51.25 ± 4.27 c | 50.00 ± 4.56 c | | 50.00 ± 4.56 c | | 50.00± 4.56 c | | 42.50 ± 4.33 c | |  |  |
| F-test | | | F-value  P-value | *F*_5, 15_= 79.01  < 0.0001 | *F*_5, 15_= 71.00  < 0.0001 | *F*_5, 15_= 83.58  < 0.0001 | *F*_5, 15_= 91.75  < 0.0001 | | *F*_5, 15_= 167.77  < 0.0001 | | *F*_5, 15_= 104.32  < 0.0001 | | *F*_5, 15_= 107.17  < 0.0001 | |  |  |
| Lab assay-4 | Pure Bt | | | | 2.50 ± 1.44 a | 0.00 ± 0.00 a | 0.00 ± 0.00 a | 0.00 ± 0.00 a | | 0.00 ± 0.00 a | | 0.00 ± 0.00 a | | 0.00 ± 0.00 a | |  |
|  | RIB | A1-Bt | | | 11.25 ± 4.27 b | 1.25 ± 1.25 a | 0.00 ± 0.00 a | 0.00 ± 0.00 a | | 0.00 ± 0.00 a | | 0.00 ± 0.00 a | | 0.00 ± 0.00 a | |  |
|  |  | A3-Bt | | | 7.50 ± 1.44 ab | 1.25 ± 1.25 a | 0.00 ± 0.00 a | 0.00 ± 0.00 a | | 0.00 ± 0.00 a | | 0.00 ± 0.00 a | | 0.00 ± 0.00 a | |  |
|  |  | B-Bt | | | 3.75 ± 2.39 a | 1.25 ± 1.25 a | 0.00 ± 0.00 a | 0.00 ± 0.00 a | | 0.00 ± 0.00 a | | 0.00 ± 0.00 a | | 0.00 ± 0.00 a | |  |
|  |  | Refuge | | | 81.25 ± 4.27 c | 56.25 ± 7.18 b | 35.00 ± 5.40 b | 16.25 ± 4.27 b | | 10.00 ± 3.54 b | | 6.25 ± 2.39 b | | 3.75 ± 1.25 b | |  |
|  | Pure non-Bt | | | | 73.75 ± 4.27 c | 51.25 ± 2.39 b | 42.50 ± 2.50 c | 40.00 ± 3.53 c | | 40.00 ± 3.53 c | | 38.75 ± 3.15 c | | 36.25 ± 3.15 c | |  |
|  | F-test | | F-value | | *F*_5, 15_= 98.32 | *F*_5, 15_= 84.59 | *F*_5, 15_= 174.40 | *F*_5, 15_= 94.58 | | *F*_5, 15_= 45.40 | | *F*_5, 15_= 65.72 | | *F*_5, 15_= 96.79 | |  |
|  |  | | P-value | | < 0.0001 | < 0.0001 | < 0.0001 | < 0.0001 | | < 0.0001 | | < 0.0001 | | < 0.0001 | |  |

^a^ Treatment mean in Lab assay-1 was based on 60 larvae, while it was based on 80 larvae for the rest three assays. Means in a column within a lab assay followed by a different letter were significantly different (Tukey’s HSD test, α=0.05). Pure Bt: pure Bt maize planting; pure non-Bt: pure non-Bt maize planting; RIB refuge: the refuge plants in the RIB planting; A1-Bt: the Bt plants immediately adjacent and within the same row as the refuge plant in RIB planting; A3-Bt: the 3^rd^ Bt plants on both sides of the refuge plant in the same row in RIB planting, and B-Bt: the closest Bt plants on both sides of the refuge plant in the two adjacent rows in RIB planting.

^b^ Lab assay-1 contained three replications with 8-10 ears per replication, while the rest three assays consisted of four replications with 8-10 ears per replication.

^c^ NTP: neonate to pupa.

^d^ NTA: neonate to adult.
