# Supplementary material for: A Challenge for the Seed Mixture Refuge Strategy in Bt Maize: Impact of Cross-Pollination on an Ear-Feeding Pest, Corn Earworm
Source: PLoS One. 2014 Nov 19;9(11):e112962. doi: 10.1371/journal.pone.0112962 (PMC4237366; doi:10.1371/journal.pone.0112962)
Supplement: Table S4 — Lab assay on development index, pupal weight, and development time (mean ± sem) of H. zea on ears of SmartStax Bt and non-Bt maize plants in three planting patterns. (DOCX) [file pone.0112962.s006.docx]

**Table S4.** Lab assay on development index, pupal weight, and development time (mean ± sem) of *H. zea* on ears of SmartStax Bt and non-Bt maize plants in three planting patterns ^a^.

| Trial | Ears | | | | | Development index^b^ | | | | Pupal mass and development duration | | | |
| --- | --- | --- | --- | --- | --- | --- | --- | --- | --- | --- | --- | --- | --- |
|  |  | | | | | 6-d | 9-d | 12-d | 15-d | No. pupa | Pupal mass (mg/pupa) | NTP^c^ | NTA^d^ |
| Lab assay-1 | Pure Bt | | | | | 2.00 a  2.00 a | ---  --- | ---  --- | ---  --- | --- | --- | --- | --- |
|  | RIB | | | | A1-Bt |  |  |  |  | --- | --- | --- | --- |
|  |  | | | | A3-Bt | 2.00 a | --- | --- | --- | --- | --- | --- | --- |
|  |  | | | | B-Bt | 2.00 a | --- | --- | --- | --- | --- | --- | --- |
|  |  | | | | Refuge | 2.68 ± 0.11 b | 3.32 ± 0.09 a | 4.04 ± 0.20 a | 4.67 ± 0.22 a | 4 | 264.2 ± 29.2 a | 20.75 ± 1.60 b | 32.50 ± 3.50 b |
| Pure non-Bt | | | | | 3.17 ± 0.07 c | 5.02 ± 0.21 b | 6.00 ± 0.10 b | 6.66 ± 0.13 b | 22 | 409.7 ± 7.4 b | 14.55 ± 0.38 a | 26.94 ± 0.37 a |  |
| F-test | | F-value  P-value | | | *F*_5, 3_= 30.88  0.009 | *F*_1, 2_= 379.76  0.003 | *F*_1, 2_= 62.25  0.016 | *F*_1, 2_= 49.79  0.020 |  | *F*_1, 22_= 55.19  < 0.0001 | *F*_1, 22_= 26.32  <0.0001 | *F*_1, 16_= 13.08  0.002 |  |
| Lab assay-2 | Pure Bt | | | | | 2.00 a | --- | --- | --- | --- | --- | --- | --- |
|  | RIB | A1-Bt | | | | 2.00 ± 0.00 a | --- | --- | --- | --- | --- | --- | --- |
|  |  | A3-Bt | | | | 2.00 a | --- | --- | --- | --- | --- | --- | --- |
|  |  | B-Bt | | | | 2.00 ± 0.00 a | 2.00 a  3.19 ± 0.15 b | 3.00 a | --- | --- | --- | --- | --- |
|  |  | Refuge | | | | 2.39 ± 0.13 a |  | 3.79 ± 0.17 a | 4.22 ± 0.19 a | 5 | 283.3 ± 30.6 a | 21.00 ± 1.30 b | 32.40 ± 1.44 b |
|  | Pure non-Bt | | | | | 3.41 ± 0.10 b | 5.04 ± 0.08 c | 5.89 ± 0.06 b | 6.51 ± 0.15 b | 32 | 437.3 ± 11.3 b | 14.88 ± 0.33 a | 26.76 ± 0.34 a |
|  | F-test | | | F-value | | *F*_5, 6_= 51.98 | *F*_2, 3_= 101.13 | *F*_2, 3_= 72.84 | *F*_1, 3_= 76.18 |  | *F*_1, 32_= 23.93 | *F*_1, 32_= 32.96 | *F*_1, 29_= 28.31 |
|  |  |  |  | P-value | | < 0.0001 | 0.002 | 0.003 | 0.003 |  | < 0.0001 | < 0.0001 | 0.002 |
| Lab assay-3 | Pure Bt | | | | | 2.00 ± 0.00 a | 2.00 a | --- | --- | --- | --- | --- | --- |
|  | RIB | A1-Bt | | | | 2.00 ± 0.00 a | 2.50 ± 0.50 a | 4.00 b | --- | --- | --- | --- | --- |
|  |  | A3-Bt | | | | 2.00 ± 0.00 a | 2.00 a | 3.00 a | --- | --- | --- | --- | --- |
|  |  | B-Bt | | | | 2.00 ± 0.00 a | 3.00 ab  3.35 ± 0.06 b | --- | --- | --- | --- | --- | --- |
|  |  | Refuge | | | | 2.60 ± 0.03 b |  | 4.35 ± 0.12 c | 5.14 ± 0.15 a | 4 | 309.0 ± 33.3 a | 17.75 ± 1.31 b | 29.00 ± 2.08 b |
|  | Pure non-Bt | | | | | 3.64 ± 0.20 c | 5.43 ± 0.22 c | 6.49 ± 0.18 d | 6.95 ± 0.03 b | 40 | 412.4 ± 6.8 b | 12.85 ± 0.21 a | 23.62 ± 0.28 a |
|  | F-test | | | F-value | | *F*_5, 10_= 361.40 | *F*_5, 4_= 26.15 | *F*_3, 3_= 1041.87 | *F*_1, 3_= 115.09 |  | *F*_1, 39_= 25.94 | *F*_1, 39_= 37.47 | *F*_1, 32_= 23.27 |
|  |  |  |  | P-value | | < 0.0001 | 0.004 | < 0.0001 | 0.002 |  | < 0.0001 | < 0.0001 | < 0.0001 |
| Lab assay-4 | Pure Bt | | | | | 2.00 ± 0.00 a | --- | --- | --- | --- | --- | --- | --- |
|  | RIB | A1-Bt | | | | 2.00 ± 0.00 a | 3.00 b | --- | --- | --- | --- | --- | --- |
|  |  | A3-Bt | | | | 2.00 ± 0.00 a | 3.00 b | --- | --- | --- | --- | --- | --- |
|  |  | B-Bt | | | | 2.00 ± 0.00 a | 2.00 a  3.53 ± 0.05 c | --- | --- | --- | --- | --- | --- |
|  |  | Refuge | | | | 2.48 ± 0.04 b |  | 4.53 ± 0.18 a | 5.33 ± 0.14 a | 5 | 277.5 ± 16.2 a | 17.40 ± 0.60 b | 29.00 ± 1.53 b |
|  | Pure non-Bt | | | | | 3.74 ± 0.07 c | 5.71 ± 0.04 d | 6.24 ± 0.08 b | 6.94 ± 0.03 b | 31 | 393.4 ± 7.2 b | 13.26 ± 0.25 a | 24.62 ± 0.30 a |
|  | F-test | | | F-value | | *F*_5, 10_= 361.40 | *F*_4, 3_= 522.12 | *F*_1, 3_= 58.41 | *F*_1, 3_ = 149.21 |  | *F*_1, 31_= 45.16 | *F*_1, 31_= 30.08 | *F*_1, 27_= 15.60 |
|  |  |  |  | P-value | | < 0.0001 | 0.0001 | 0.005 | 0.001 |  | < 0.0001 | < 0.0001 | 0.0005 |

^a^ Pure Bt: pure Bt maize planting; Pure non-Bt: pure non-Bt maize planting; RIB refuge: the refuge plants in the RIB planting; A1-Bt: the Bt plants immediately adjacent and within the same row as the refuge plant in RIB planting; A3-Bt: the 3^rd^ Bt plants on both sides of the refuge plant in the same row in RIB planting, and B-Bt: the closest Bt plants on both sides of the refuge plant in the two adjacent rows in RIB planting. Sample size for each mean for measuring development index was 16-66 larvae for pure non-Bt and RIB refuge and 1-9 larvae for Bt plants. Means in a column within a lab assay followed by a different letter were significantly different (Tukey’s HSD test, α=0.05).

^b^ Insect development were converted to development index: 1= 1st instar, 2= 2nd instar, …, 6= 6th instar, 7= pupal stage.

^c^ NTP: neonate-to-pupa development time (d).

^d^ NTA: neonate-to-adult development time (d).
