# Supplementary material for: A Challenge for the Seed Mixture Refuge Strategy in Bt Maize: Impact of Cross-Pollination on an Ear-Feeding Pest, Corn Earworm
Source: PLoS One. 2014 Nov 19;9(11):e112962. doi: 10.1371/journal.pone.0112962 (PMC4237366; doi:10.1371/journal.pone.0112962)
Supplement: Table S5 — Field-plus-lab assay on pupal mass (mean ± sem) of H. zea on ears of RIB refuge and pure non-Bt plants. (DOCX) [file pone.0112962.s007.docx]

**Table S5.** Field-plus-lab assay on pupal mass (mean ± sem) of *H. zea* on ears of RIB refuge and pure non-Bt plants ^a^.

| Ears | | Total No. pupa | Pupal weight (mg) |
| --- | --- | --- | --- |
| RIB refuge | | 41 | 334.7 ± 13.6 a |
| Pure non-Bt | | 227 | 433.2 ± 5.1 b |
| F-test | F-value | --- | *F*_1, 2_= 52.14 |
|  | P-value | --- | 0.02 |

^a^ Means in a column followed by a different letter were significantly different (Tukey’s HSD test, α=0.05). RIB refuge: the refuge plants in the RIB planting, and pure non-Bt: pure non-Bt maize planting.
